# Detecting Coal Pulverizing System Anomaly Using a Gated Recurrent Unit and Clustering

**DOI:** 10.3390/s20113271

**Published:** 2020-06-08

**Authors:** Zian Chen, Zhiyu Yan, Haojun Jiang, Zijun Que, Guozhen Gao, Zhengguo Xu

**Affiliations:** 1 College of Control Science and Engineering, Zhejiang University, Hangzhou 310000, China; 3170103156@zju.edu.cn (Z.C.); zy_yan@zju.edu.cn (Z.Y.); 21932056@zju.edu.cn (G.G.); xzg@zju.edu.cn (Z.X.); 2 College of Computer Science and Technology, Zhejiang University, Hangzhou 310000, China; 3170104174@zju.edu.cn

**Keywords:** anomaly detection, gated recurrent unit, temporal, clustering

## Abstract

The coal pulverizing system is an important auxiliary system in thermal power generation systems. The working condition of a coal pulverizing system may directly affect the safety and economy of power generation. Prognostics and health management is an effective approach to ensure the reliability of coal pulverizing systems. As the coal pulverizing system is a typical dynamic and nonlinear high-dimensional system, it is difficult to construct accurate mathematical models used for anomaly detection. In this paper, a novel data-driven integrated framework for anomaly detection of the coal pulverizing system is proposed. A neural network model based on gated recurrent unit (GRU) networks, a type of recurrent neural network (RNN), is constructed to describe the temporal characteristics of high-dimensional data and predict the system condition value. Then, aiming at the prediction error, a novel unsupervised clustering algorithm for anomaly detection is proposed. The proposed framework is validated by a real case study from an industrial coal pulverizing system. The results show that the proposed framework can detect the anomaly successfully.

## 1. Introduction

The coal pulverizing system is an important auxiliary system in thermal power generation systems whose function is to prepare qualified pulverized coal for combustion according to the requirements of power generation [1]. Keeping the coal pulverizing system working in normal condition is crucial to ensure the safety and economy of thermal power generation systems. Therefore, prognostics and health management (PHM) is an effective approach to assess the reliability of the coal pulverizing system [2]. As an important part of PHM, anomaly detection aims to monitor the coal pulverizing system working condition based on condition monitoring (CM) data and to detect whether the coal pulverizing system works in normal condition or not [3].

In general, the anomaly detection approaches can be classified into two categories: model-based approaches and data-driven approaches [4]. Among them, model-based approaches require an accurate mathematical model to describe the system working condition. However, the coal pulverizing system is a complex system, and to construct an accurate mathematical model is by no means an easy task [5]. In this case, the data-driven approach, which makes use of historical CM data to enable knowledge discovery and precise decision making, receives much more attention [6].

Among the data-driven approaches, the signal processing and analysis approach has been widely used. The core idea of the signal processing and analysis approach is to enhance the diagnostic information and extract a health index (HI) to detect anomalies. Jin extracted the mahalanobis distance to construct an HI and employed a Box–Cox transformation to detect bearing anomalies [7]. Liu constructed an HI from the cyclostationary domain and proposed a novel support vector data description for anomaly detection [8]. Lin employs the ARIMA algorithm to predict the fault of a throttle valve [9]. Li predicts the degradation trend of rotating machinery using a particle filter algorithm [10]. Although the signal processing and analysis approach has good performance on anomaly detection, it is difficult to manually interrogate for the presence of damage, and it has the limitation of processing low-dimensional data. What’s more, the need for a lot of data may impede the efficiency [11].

Hence, machine learning techniques, which are considered as black-box models and have a great ability to deal with high-dimensional data, have attracted much more attention. Zhao proposed an improved FDA algorithm to excavate the correlation between parameters to predict the degradation fault [12]. Wu used a polynomial neural network and data fusion technology to predict the fault [13]. Choi employed a long short-term memory (LSTM) network to detect sensor faults [14]. Que proposed a XGBoost-based framework to detect anomalies in steam turbines [15]. These machine learning approaches mostly belong to supervised learning, which is sensitive to the amount and the quality of training data. In order to obtain better performance, supervised learning requires that the training data contain all working conditions and that the number of normal and abnormal data labels is similar. However, the coal pulverizing system most of the time works in healthy condition and lacks abnormal labels. Moreover, it is impractical to obtain all working conditions. Thus, novelty detection approaches are needed.

Discrepancy analysis, which is a kind of semi-supervised approach, is developed to circumvent the aforementioned problems. The discrepancy-analysis-based anomaly detection approach usually involves two modules: working condition prediction and online anomaly detection. In the working condition prediction module, the CM data in the normal condition are collected to construct a benchmark machine learning model. The model is employed to obtain the potentially high-dimensional nonlinear relationship between the CM data and key parameters. The values of key parameters are defined as the condition values, which are used to reflect the working condition. Many machine learning approaches are proposed in the working condition prediction field. Stief proposed a sensor fusion approach based on the combination of a two-stage Bayesian method and principal component analysis (PCA) for condition monitoring of induction motors [16]. Liang used the gray correlation algorithm to extract the eigenvectors of monitoring data and then used support vector regression (SVR) to monitor the condition of wind turbines [17]. Yao proposed a multi-scale convolutional neural network(CNN) to mine multiple scale features from raw acoustic signals [18]. However, these approaches only deal with CM data as discrete time points and ignore the temporal information. The coal pulverizing system is a typical dynamic system with much temporal information. In order to consider temporal information, the recurrent neural network (RNN) and its variants have been widely used. Zhao combined CNN and bi-directional LSTM to monitor machine health [19]. Qian proposed a novel condition-monitoring method for wind turbines based on LSTM algorithms [20]. However, the LSTM network has a complex architecture and a large calculation [21]. As an improvement on LSTM, gated recurrent unit (GRU) can solve the problem of condition prediction in time series with a smaller calculation [22].

In the online detection module, the predicted condition value is obtained by employing the trained benchmark and online CM data. Then, the discrepancy between the prediction and real condition values can be obtained. Since the benchmark model only learns the potential relationship in the normal working condition, the prediction error would gradually increase when there are anomalies. Therefore, signal processing technology is used to analyze the deviation signal and detect anomalies. The threshold approach is often used to determine whether an anomaly occurs. Cheng employed a threshold from integrity requirement to detect space anomalies [23]. Hassan employed a block maxima method to determine an accurate threshold [24]. However, the threshold needs to be determined by a priori knowledge, and an inappropriate threshold also affects the accuracy of abnormal detection. Therefore, the clustering algorithm has received attention. Many unsupervised clustering algorithms have been developed. Yiakopoulos used K-means for automated diagnosis of defective rolling bearings [25]. He proposed an improved density-based spatial clustering of applications with noise (DBSCAN) algorithm for fault detection [26]. Alex proposed a novel density-based fast clustering algorithm on the idea that cluster centers are characterized by a higher density than their neighbors and by a relatively large distance from points with higher densities [27]. These clustering algorithms have good performance, but they ignore temporal information.

To address the aforementioned challenges, a novel data-driven integrated framework for anomaly detection of coal pulverizing systems is proposed. The contributions are summarized as follows:

1. A GRU neural network is constructed to predict the condition value by learning temporal information of the coal pulverizing system in the normal working condition;
2. A novel unsupervised clustering algorithm is proposed to deal with the discrepancy and detect the anomalies according to the clustering results;
3. A real case from an industrial coal pulverizing system is leveraged to evaluate the performance of the proposed framework.

The rest of this paper is organized as follows. Section 2 gives a concise description of the coal pulverizing system and GRU network. Section 3 describes the proposed approach, including the GRU neural network model for condition prediction and a novel unsupervised clustering algorithm used for anomaly detection. Section 4 reports the case study results for an industrial coal pulverizing system. Section 5 concludes this work and declares our future work.

## 2. Background and Related Work

### 2.1. Coal Pulverizing System

Thermal power generation systems convert the heat energy generated during combustion into electric energy through the power generation unit. In the production process, fossil fuels are burned to heat the water into high-temperature and high-pressure steam, which is used to drive the generator for power generation.

**Figure 1:**
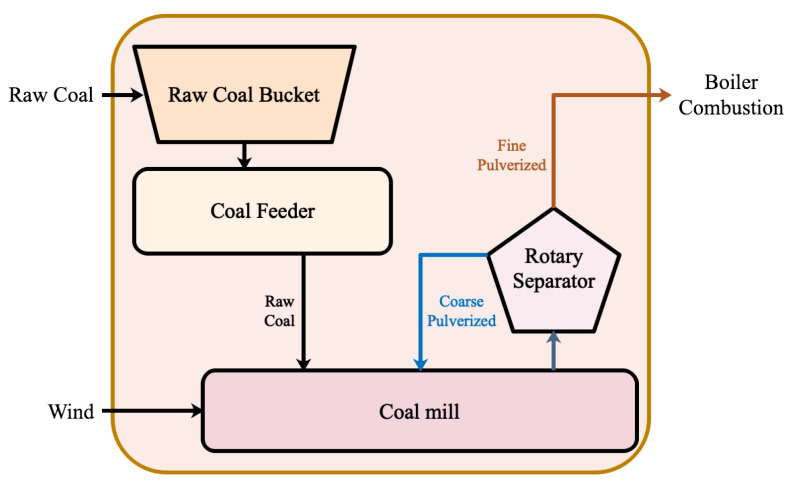
Schematic of a coal pulverizing system.

Among the fossil fuels, due to its high combustion efficiency, wide adaptability, and rapid load response, pulverized coal is the main fossil fuel for large thermal power systems. In order to ensure the stability of combustion and the economy of production, fine and dry pulverized coal is required. The main function of the coal pulverizing system in the thermal power system is to prepare qualified pulverized coal, transfer the pulverized coal to the boiler system for combustion, and discharge the unmilled coal in the form of stone coal. A typical coal pulverizing system is shown in Figure 1.

A typical coal pulverizing system includes a raw coal bucket, coal feeder, coal mill, rotary separator, and the corresponding pipeline. The raw coal is transported to the raw coal bucket by the conveyor belt. Then it goes into the coal feeder. The coal feeder controls the amount of coal through the speed to satisfy the demand of the power generation load. The raw coal is piped into the coal mill. In the coal mill, the raw coal is milled into pulverized coal. The pulverized coal is carried into the rotary separator by wind. The fine pulverized coal is sent to the boiler system, and the coarse pulverized coal is returned to the coal mill for further grinding.

The coal pulverizing system is composed of several subsystems. Each subsystem is made up of several multiple devices, and subsystems are also interconnected. Therefore, it is difficult to build mathematical models to describe the operation condition and detect anomalies for the coal pulverizing system. Process monitoring data, which contains the system condition information, can be used to assess the operating condition and detect whether there is an anomaly or not [15]. In order to detect the operating condition of the coal pulverizing system more accurately, it is necessary to obtain enough information from multiple process measure data, such as temperature, current, speed, etc. Generally, the coal pulverizing system is a nonlinear and complex system.

**Figure 2:**
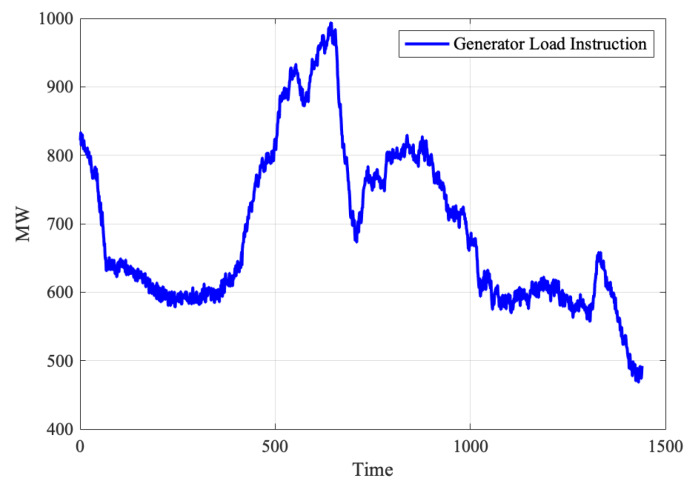
Schematic of the generator load instruction.

Due to the requirements of production, the power generation load of the thermal power system is often in a changing condition. As shown in Figure 2, it can be found that the power generation load varies over time. With the constant change of the power generator load, the amount of pulverized coal in the coal pulverizing system should also change accordingly. Therefore, the coal pulverizing system is a typical dynamic system. When analyzing the coal pulverizing system, the process monitoring data should be consider as a time series, and it is necessary to use the time-series approach to monitor the operation condition.

### 2.2. Gated Recurrent Unit

Recently, the machine learning approach has attracted more and more attention for its ability to process high-dimensional and nonlinear data. With the development of machine learning and computing capacity, important breakthroughs have been made in many problems, including sequence-to-sequence learning tasks [28]. The recurrent neural network (RNN), which can combine historical information with the current output, is significant for dealing with the time-series problem [29]. However, due to the vanishing gradient problem during backpropagation, the standard RNN can only keep short-term memory [30]. To overcome this problem, the LSTM network was first presented by introducing a gates function in the design of the transition function [31].

**Figure 3:**
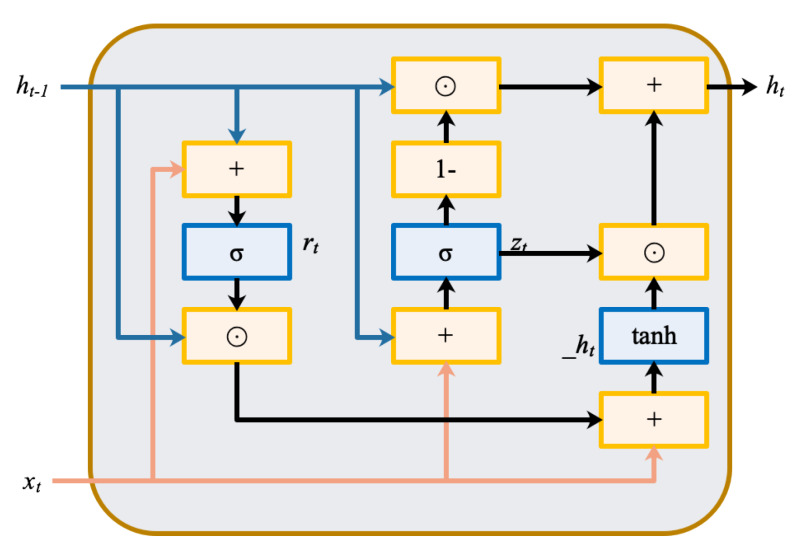
Schematic of the generator load instruction.

The GRU, proposed by Choi [32], is an improved form of LSTM. Compared with LSTM, the GRU has a smaller architecture, a lower computation cost, and comparable performance [33]. Figure 3 shows the block architecture for an unrolled GRU [29]. The GRU consists of a reset gate $r_{t}$ and an update gate $z_{t}$, where *t* is the time point. $x_{t}$ is the input vector at time *t*, $h_{t-1}$ is the recurrent GRU block output at time t−1, and $h_{t}$ is the current output at time *t*. ${\tilde{h}}_{t}$ is the output candidate of the GRU block at time *t*.

The reset gate $r_{t}$ controls how much information is to be transformed from $h_{t-1}$ to the ${\tilde{h}}_{t}$. The update gate $z_{t}$ decides how much the block updates its activation at time *t*. The formula for forward propagation is

(1) $$r_{t}=\sigma \left(W_{rx}x_{t}+W_{rh}h_{t-1}+b_{r}\right);$$

(2) $$z_{t}=\sigma \left(W_{zx}x_{t}+W_{zh}h_{t-1}+b_{z}\right);$$

(3) $${\tilde{h}}_{t}=\tanh\left(W_{\tilde{h}x}x_{t}+W_{\tilde{h}h}\left(r_{t}⊙h_{t-1}\right)+b_{\tilde{h}}\right);$$

(4) $$h_{t}=\left(1-z_{t}\right)⊙h_{t-1}+z_{t}⊙{\tilde{h}}_{t},$$

where $W_{rx}$, $W_{zx}$, and $W_{\tilde{h}x}$ are the weights for $x_{t}$. $W_{rh}$, $W_{zh}$, and $W_{\tilde{h}h}$ are the weights for $h_{t-1}$. $b_{r}$, $b_{z}$, and $b_{\tilde{h}}$ are the bias. More details on the GRU can be found in [34].

From Equations (1)–(5), it can be found that the the recurrent GRU block output $h_{t}$ is not only related to the current CM data $x_{t}$ but is also related to the recurrent $h_{t-1}$. That is, some valid information from the past time is transferred to the current moment. Due to the particularity of the GRU structure, the correlation between time steps is considered and a regression relationship is established with the final output. Therefore, it can effectively process the temporal information.

## 3. Methods

### 3.1. Framework

**Figure 4:**
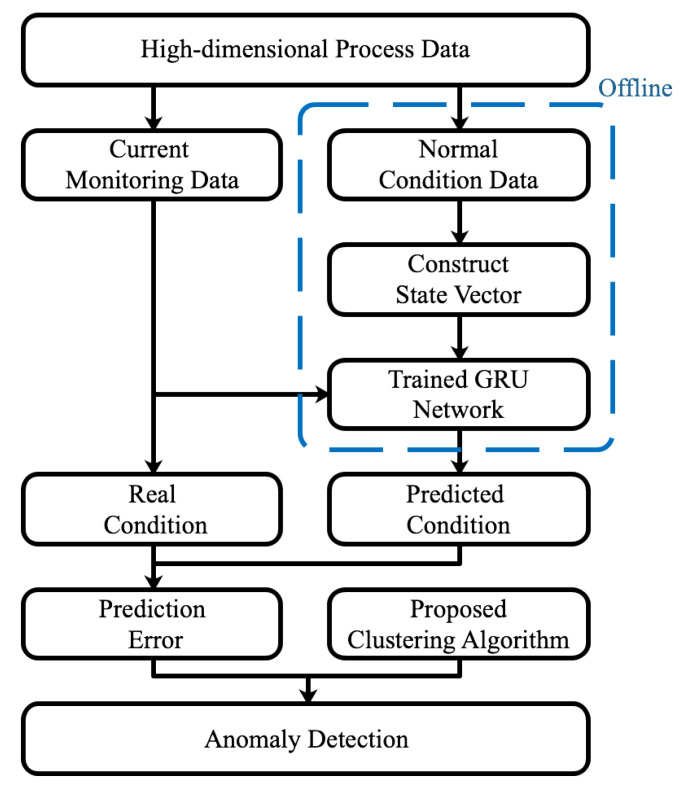
Schematic of the proposed data-driven approach.

This paper proposes a data-driven integrated framework for anomaly detection in coal pulverizing systems. The proposed framework contains two modules. First, a GRU neural network is constructed to predict the condition value. Then, a novel unsupervised clustering algorithm is proposed to automatically detect anomalies according to the prediction errors. The schematic of the proposed data-driven anomaly detection approach is shown in Figure 4.

The major steps of the proposed framework are listed as follows.

(1) The high-dimensional time-series data in the normal working condition are selected to construct the training set.
(2) Construct the neural network model and train it with the training set.
(3) The current monitoring data are input into the trained neural network to obtain the predicted working condition.
(4) The prediction error is calculated by using the real condition value and the predicted condition value.
(5) A novel unsupervised clustering algorithm is used to cluster prediction errors and detect anomalies.

Among these steps, steps 1 and 2 are realized offline; steps 3, 4, and 5 are constructed online.

### 3.2. Condition Value Prediction

In this module, a GRU neural network is constructed to predict the condition value of the system. The GRU neural network has the ability to learn the relationship between the past condition value and the current condition value. When training the GRU neural network, the data for the normal working condition are selected as the training set. Through training, it can be considered that the neural network model captures and learns the system behavior information in the normal working condition.

As a complex dynamic and nonlinear system, the coal pulverizing system may obtain multiple temporal monitoring variables at each time point. Different process monitoring data may have different scales, so process monitoring data should be normalized to eliminate the influence of scales. The normalization technique is as follows:

(5) $$c=\left(G-G_{min}\right)/\left(G_{max}-G_{min}\right),$$

where G stands for one feature in multidimensional process monitoring data, $G_{min}$ is the minimum value, and $G_{max}$ is the minimum value in G.

Therefore, the normalized temporal variables are transformed to a vector,

(6) $$C=\left\{c_{1},c_{2},\ldots ,c_{T}\right\},$$

where $c_{t}\in R^{m}$ in the vector C is a high-dimensional vector $\left\{c_{t}^{1},c_{t}^{2},\ldots ,c_{t}^{m}\right\}$ whose element corresponds to each temporal variable at time step *t*, t=1,2,…,T. *T* is the length of the selected time period, which belongs to the normal working condition. *m* is the number of temporal variables.

Since the constructed neural network model has to process time-series data, the vector C has to be divided into several sequence vectors $C^{\prime }$.

(7) $$C^{\prime }=\left\{p_{1},p_{2},\ldots ,p_{T-l+1}\right\},$$

where *l* is the time step. It determines the number of past time steps before the current time point to use for condition value prediction. $p_{t}$ is the sequence vector at time point *t*,

(8) $$p_{t}=\left\{c_{t-l+1},c_{t-l+2},\ldots ,c_{t}\right\}.$$

**Figure 5:**
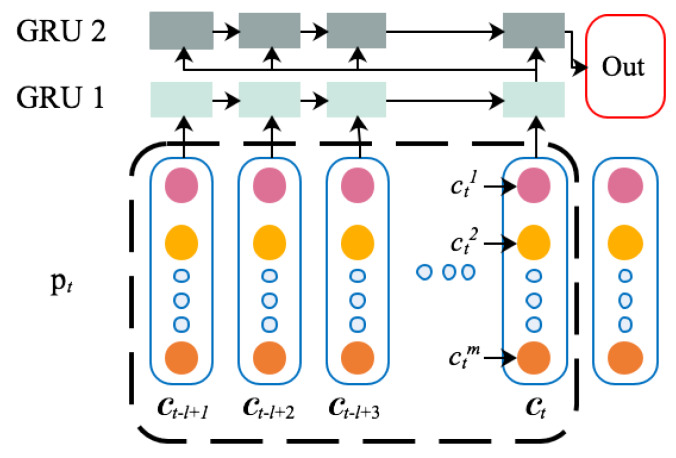
Architecture of the proposed GRU.

The sequence vector $C^{\prime }$ is the input of the neural network. The output of the neural network $y_{t}$ is the variable that can reflect the system working condition, such as power, current, and so on. When constructing the network structure, a two-layer GRU network structure is selected. The architecture of the constructed neural is shown in Figure 5.

After training the neural network, the current monitoring data are put into the trained neural network to obtain the predicted condition value ${\hat{y}}_{q}$. Then the prediction error is calculated according to $e_{q}=\left|y_{q}-{\hat{y}}_{q}\right|$, where $y_{q}$ is the real condition value at time *q*. Then, a one-dimensional vector of errors can be constructed as

(9) $$e=\left[e_{1},e_{2},\ldots ,e_{q}\right].$$

### 3.3. Anomaly Detection

In this module, the prediction error is employed to detect anomalies. When the system is in normal working condition, as the neural network has learned the behavior information in this period, the prediction error is small and within a certain range. When the system is in abnormal condition, as the behavior information has not been learned by the neural network, the prediction error will gradually increase. Therefore, the system condition can be detected by the prediction error. To evaluate whether the prediction error is in a normal working condition or not, a novel unsupervised clustering algorithm is proposed.

The temporal relationship between the current error and past errors also contains abundant information. However, traditional clustering methods only treat them as discrete points and ignore this part of the information. To solve this problem, a new unsupervised clustering algorithm is proposed.

Reconstruct the one-dimensional vector into a two-dimensional vector by adding the time point as the first dimensional value.

(10) $$e^{\prime }=\left[\left(1,e_{1}\right),\left(2,e_{2}\right),\ldots ,\left(q,e_{q}\right)\right]$$

By reconstruction, the one-dimensional error is mapped to a 2D coordinate frame with the time axis. In the traditional clustering method, the neighborhood area is usually defined as a circle, which cannot reflect the temporal information. In the proposed algorithm, the neighborhood area is changed from a circle to an ellipse. The long axis of the ellipse reflects the change rate of prediction errors at the current and past times, and the ellipse range is used to detect anomalies. Therefore, the ellipse neighborhood area contains some temporal information, which the circle neighborhood area and probability distribution function cannot extract.

At time *q*, define the point $\left(q,e_{q}\right)$ as one of the focuses of the ellipse. The other focus can be found on line $L_{q}$, where the line $L_{q}$ is determined by the current time point $\left(q,e_{q}\right)$ and the time point before the current time $\left(q-1,e_{q-1}\right)$,

(11) $$L_{q}:Y=\left(e_{q}-e_{q-1}\right)X-\left(e_{q}-e_{q-1}\right)q+e_{q}.$$

Define the major axis of the ellipse as 2a and the focal length of the ellipse as 2c. The other focus of the ellipse $\left(q^{\prime },e_{q}^{\prime }\right)$ can be determined as

(12) $$\{\begin{array}{c} \sqrt{{\left(q-q^{\prime }\right)}^{2}+{\left(e_{q}-e_{q}^{\prime }\right)}^{2}}=2c,\\ \;\left(q^{\prime },e_{q}^{\prime }\right)\in L_{q}. \end{array}$$

**Figure 6:**
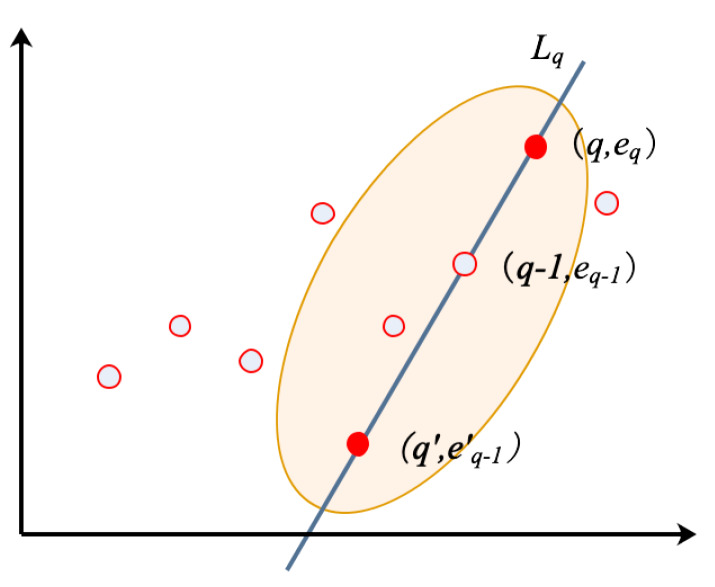
Schematic of the novel unsupervised clustering algorithm.

Actually, there are two points that satisfy the requirement. Considering the time characteristics, at time step *q*, we only consider the time before time *q*, that is, $q^{\prime }<q$. The schematic diagram of the proposed method is shown in Figure 6.

When the two focuses are determined, it can determine whether the prediction error before time point *p* is in the ellipse, and the cluster can be determined according to the number of time points in the ellipse. The specific algorithm is shown in Algorithm 1.

When the system is working in the normal working condition, the prediction error is small and fluctuates in a small range. Therefore, the prediction errors in the normal working condition belong to the normal cluster through the proposed unsupervised clustering algorithm. When the system is working in an abnormal working condition, the prediction errors gradually increase, so they are in abnormal clusters. In general, there are isolated points between different clusters. Sometimes, these isolated points are caused by the existence of noise and fluctuations in operating conditions. In this case, misjudgment will occur if only a single isolated point is used to judge. In order to avoid this problem, several continuous isolated points are selected as the criterion. At the same time, the selection of too many isolated points will affect the algorithm’s discrimination time. Therefore, three continuous isolated points are selected as the criteria to detect whether the system is transformed from the normal condition to abnormal condition.
**Algorithm 1**  The novel unsupervised clustering algorithmRequire: $e^{\prime }$: Two-dimensional error vector *a*: The major axis of the ellipse *c*: The focal length of the ellipse MinPts: Neighborhood density thresholdOutput: Clustering resultsSteps: 1: At time *q*, obtain the point $D_{q}$:$\left(q,e_{q}\right)$; 2: Calculate $l_{q}$ based on $\left(q,e_{q}\right)$ and $\left(q-1,e_{q-1}\right)$; 3: Find the point $D_{q}^{\prime }$:$\left(q^{\prime },q_{t}^{\prime }\right)$:   If ${∥D_{q}D_{q}^{\prime }∥}_{2}=2c$ and $D_{t}^{\prime }\in L_{q}$ then    If $q^{\prime }<q$ then     $D_{q}^{\prime }$ is the other focal point 4: Determine whether MinPts time steps before *q* are in the elliptic domain:   n = 0   While i<MinPts:    If ${∥D_{q}D_{q-i}∥}_{2}+{∥D_{t}^{\prime }D_{t-i}∥}_{2}<=2a$ then     n = n + 1; 5: Determine the cluster of $D_{q}$ belongs to:   If n==MinPts then    If $D_{q-1}\in ClusterA$ then     $D_{q}$ is in ClusterA;    Else $D_{q-1}$ is an isolated point then     $D_{q}$ is in ClusterB;   Else     $D_{q}$ is an isolated point.

## 4. Results and Discussion

A case from a real industrial coal pulverizing system is used to validate the proposed approach. The CM data comes from the distributed control system (DCS) in the industrial field. The CM data are selected every 5 s, and the value is defined as the average within 5 s. The multidimensional process-monitoring data in subsystems contain characteristic information that can reflect the operation condition of the coal pulverizing system. The selected features are list in Table 1. It needs to be noted that for one CM feature, there may be multiple sensors. For example, there are four sensors that measure the bearing temperature, so there are four signals for this feature. Moreover, the process-monitoring data include the whole process from normal condition to abnormal shutdown.

In the case study, the anomaly of the coal pulverizing system is coal plugging. With the coal plugging, the amount of raw coal transfer from coal feeder to coal mill is reduced. It will decrease the coal mill output, and in the case of constant voltage, the coal mill current appears to decline fast. The degradation of the coal pulverizing system is a rapid degradation process. During degradation, some other features will change accordingly, such as the decline of the powder–air mixture pressure due to the lack of powder. In this case, the coal mill current is selected as the key parameter. Through learning, the prediction error between the predicted coal mill current and the real coal mill current by the GRU model at normal time is small. When the anomaly occurs, the error increases gradually. At this time, the proposed clustering method can be used to detect the anomaly.

**Figure 7:**
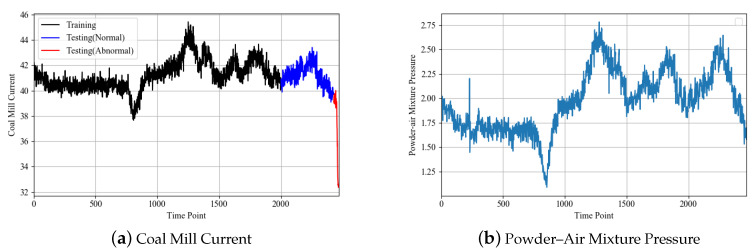
Schematic of features.

In the data set, the coal mill current is selected as the output of the GRU model, as the coal mill current can most intuitively reflect the working condition of the coal pulverizing system. Other features mentioned in Table 1 are chosen to construct the input vector. The data set contains 2451 time points. The data from the first 2000 time points are selected to build the training set. It should be noted that during this time, the coal pulverizing system is working in normal condition. The remaining 451 time points are selected as the testing set, in which there was an anomaly occurring at time point 427 that eventually led to the shutdown. The schematic of the coal mill current and the typical input feature, the powder–air mixture temperature, are shown in Figure 7.

**Figure 8:**
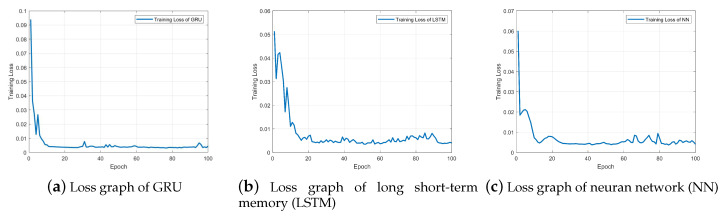
Comparison of training performance.

The training set was used to train the neural network model. The experiments were conducted on a Windows laptop with 16 G of RAM and four Intel i5 CPUs. The programming tool in this work was Python 3 using pytorch library. The ability of a single GRU model to extract temporal information is limited, so the method of stacking GRUs was adopted to improve the ability to extract temporal information. In this case study, a two-layer stacked GRU model was employed. The model structure was GRU(50)–GRU(50)–Dense(20)–Dense(1). In order to compare the performance, the LSTM network and feed-forward neural network (NN) were chosen for comparison. The LSTM network adopts the same network structure as the GRU model, i.e., LSTM(50)–LSTM(50)–Dense(20)–Dense(1). The NN model has the structures of 50–100–50–1. The training performance is shown in Figure 8.

From Figure 8, it can be seen that the GRU has the fastest rate of convergence. Compared with LSTM, GRU has only two gate structures, which are relatively simple and require less calculation for training, so it has a fast convergence rate. The training time of the GRU is 84.78 s, and the training time of LSTM is 100.96 s. Compared with the NN, the GRU finally converges to about 0.0036 with less fluctuation. NN fluctuates greatly, which is mainly due to its simple structure and limited ability to fit high-dimensional sequential data.

**Figure 9:**
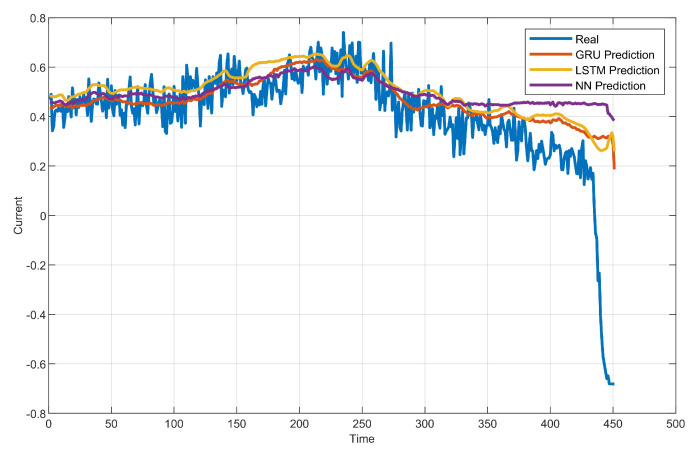
Schematic of experiment results.

After training, the testing set is used to test. The results are shown in Figure 9. It can be seen that the real coal mill current signal fluctuates up and down within a small range in the normal working condition (the first 427 time points). The tendency of the current signal is generally in a stable state. However, in the later period (427–451 time points), there is an anomaly in the coal pulverizing system, and the current showed an obvious downward trend as shown in Figure 7a.

In the normal working period of testing, the predicted condition value is close to the real condition value. This is because in the training process, the process-monitoring data containing the normal working characteristics information are used to train the constructed neural network model. After training, the neural network model has learned the behavior characteristics of the normal working condition. In the former period of testing, the coal pulverizing system is still in normal working condition, so the prediction is accurate.

In the abnormal working period of testing, the predicted condition value deviates from the real condition value. This is because during the training process, the abnormal working condition characteristics information has not been learned. Therefore, when an abnormality occurs, the output of the neural network cannot predict the current condition value well. With the continuous degradation, the deviation between the predicted current value and the real current value is larger and larger.

Comparing the constructed GRU network with LSTM, it can be found that the GRU network has a similar performance to LSTM. This shows that compared with LSTM, the GRU has a more reduced structure, faster training speed, and similar nonlinear fitting ability. Compared with the NN, the performance is much better. This is because, compared with the NN, the GRU takes into account the temporal information and the correlation between the current and past time points. More information makes the predictions of the GRU more accurate.

**Figure 10:**
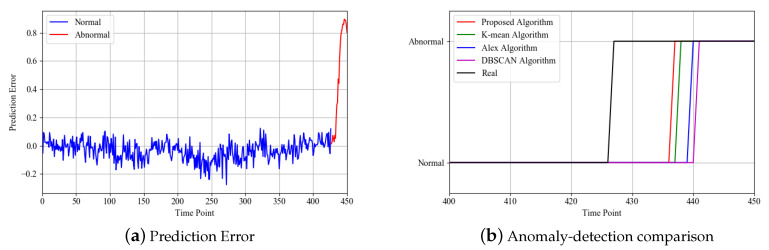
Anomaly detection results.

In order to observe the variation of the prediction error more clearly, the variation of the prediction error with time points is shown in Figure 10a. It can be seen that the prediction error is around 0 in the normal working condition, which indicates that the predicted condition value is close to the real condition value. When an anomaly occurs, the prediction error gradually increases. An obvious upward trend can be obtained.

After predicting the condition value, the prediction errors are used to detect anomalies. When the coal pulverizing system is in normal condition, the prediction error is small and can be classified into normal clusters. When the coal pulverizing system is in abnormal working condition, the prediction error gradually increases. If the clustering results do not belong to the normal cluster, the coal pulverizing system can be considered as being in abnormal working condition. The proposed algorithm is compared with Alex’s proposed algorithm, the K-means and DBSCAN algorithm. The comparison results are shown in Figure 10b.

From Figure 10b, it can be seen that compared with the real curve, the used algorithms have significant lag. This is mainly because at the initial stage of the occurrence of anomalies, the characteristics of the anomalies are not obvious enough, resulting in a small gap between them and the normal period, as shown in Figure 10a. Compared with the classical clustering method, the proposed algorithm was the first to detect the anomaly. This is mainly because the proposed method takes into account the temporal information and thus obtains more accurate anomaly detection results.

## 5. Conclusions

This paper has proposed a data-driven integrated framework for anomaly detection of coal pulverizing systems. The proposed framework includes two modules: condition value prediction and anomaly detection. A GRU model is employed to predict the condition value and a novel unsupervised clustering algorithm is constructed to detect anomalies. An industrial coal pulverizing system is leveraged to evaluate the performance. The GRU-based network can effectively extract the temporal information and predict the condition value. The proposed clustering algorithm can detect anomalies earlier than the traditional algorithms.

In the current study, the GRU model adopts a traditional structure without optimization. The proposed unsupervised clustering method has good performance on anomaly detection, but it cannot detect anomalies in the early stage. The proposed clustering algorithm has the limitation of a slow degradation process. Therefore, in future work, the GRU model should be optimized to extract the temporal information more accurately. The proposed algorithm should be optimized to improve the accuracy of anomaly detection in the early stage and extend it to other degradation processes.

## Figures and Tables

**Table 1:** The selected condition monitoring (CM) features.

Feature Name			
Coal Feeder Current	Coal Mill Current	Rotary Separator Current	Air Temperature
Powder–Air Mixture Pressure	Bearing Temperature	Coal–Air Baffle Position	Hot-Air Baffle Position
Lubricating Oil Pressure	Oil Tank Temperature	Lubricating Oil Temperature	Rotary Separator Speed
Seal Wind Pressure	Grinding Bowl Pressure	Primary Air Volume	Primary Air Flow
Instantaneous Coal Feed	Coal Feeder Motor Speed	Coal Feed Accumulation	Generator Phase Voltage
